# Supplementary material for: Polyketide synthase gene clusters in the ericoid mycorrhizal fungus Oidiodendron maius: genome-wide prediction, expression profiling, and functional characterization of knockout mutants
Source: Front Plant Sci. 2026 Sep 16;17:1924430. doi: 10.3389/fpls.2026.1924430 (PMC13623903; doi:10.3389/fpls.2026.1924430)
Supplement: Supplementary file 1 [file SupplementaryFile1.zip › Supplementary Materials/Supplementary Folder 1.zip!/Oidma1_AssemblyScaffolds_Repeatmasked/knownclusterblast/cluster55/jgi.p_Oidma1_138779_mibig_hits.html]

| MIBiG Protein | Description | MIBiG Cluster | MiBiG Product | % ID | % Coverage | BLAST Score | E-value |
| --- | --- | --- | --- | --- | --- | --- | --- |
| EAL89340.1 | toxin\_biosynthesis\_regulatory\_protein\_AflJ,\_putative | BGC0001403 | Trypacidin\_biosynthetic\_gene\_cluster | 41 | 99.3534482759 | 285 | 1.4e-76 |
| CBF90109.1 | toxin\_biosynthesis\_regulatory\_protein\_AflJ,\_putative\_(AFU\_orthologue;\_AFUA\_4G14550) | BGC0001271 | Emericellin\_biosynthetic\_gene\_cluster | 38 | 107.543103448 | 268 | 1.3e-71 |
| AGO59050.1 | PtaR1 | BGC0000121 | Pestheic\_acid\_biosynthetic\_gene\_cluster | 39 | 90.7327586207 | 235 | 9.6e-62 |
| ACH72906.1 | AflS | BGC0000011 | Aflatoxin/sterigmatocystin\_biosynthetic\_gene\_cluster | 38 | 85.5603448276 | 222 | 8.4e-58 |
| AAS90096.1 | AflJ | BGC0000006 | Aflatoxin\_biosynthetic\_gene\_cluster | 37 | 87.5 | 212 | 1.5e-54 |
| AAS90027.1 | AflJ | BGC0000008 | Aflatoxin\_biosynthetic\_gene\_cluster | 37 | 87.5 | 212 | 1.5e-54 |
| BAE71319.1 | possible\_transcriptional\_modulator | BGC0000004 | Aflatoxin\_biosynthetic\_gene\_cluster | 37 | 87.5 | 211 | 1.9e-54 |
| AAS90004.1 | AflJ | BGC0000007 | Aflatoxin\_biosynthetic\_gene\_cluster | 36 | 87.5 | 210 | 4.3e-54 |
| AAS90072.1 | AflJ | BGC0000010 | Aflatoxin\_biosynthetic\_gene\_cluster | 37 | 87.5 | 207 | 4.8e-53 |
| AAS90052.1 | AflJ | BGC0000009 | Aflatoxin\_biosynthetic\_gene\_cluster | 35 | 87.2844827586 | 198 | 2.2e-50 |
